# Repetition Priming in Individuals with Amnestic Mild Cognitive Impairment and Alzheimer’s Dementia: a Systematic Review and Meta-Analysis

**DOI:** 10.1007/s11065-021-09504-5

**Published:** 2021-04-25

**Authors:** Liselotte De Wit, Vitoria Piai, Pilar Thangwaritorn, Brynn Johnson, Deirdre O’Shea, Priscilla Amofa, Michael Marsiske, Roy P. C. Kessels, Nancy Schaefer, Glenn Smith

**Affiliations:** 1 Department of Clinical and Health Psychology, University of Florida, P.O. Box 100165, 32610-0165 Gainesville, FL USA; 2 Donders Institute for Brain, Cognition and Behaviour, Centre for Cognition, Radboud University, Thomas van Aquinostraat 4, 6525 GD Nijmegen, Netherlands; 3 University of Florida Health Science Center Libraries, University of Florida, SW Archer Rd, 32610 Gainesville, FL USA; 4 Donders Institute for Brain, Cognition and Behaviour, Centre for Medical Neuroscience, Department of Medical Psychology, Radboud University Medical Center, PO Box 9101, 6500 HB, Nijmegen, Netherlands

**Keywords:** Alzheimer’s disease, Mild Cognitive Impairment, Repetition priming, Priming, Implicit Memory

## Abstract

The literature on repetition priming in Alzheimer’s disease (AD) is inconsistent, with some findings supporting spared priming while others do not. Several factors may explain these inconsistencies, including AD severity (e.g., dementia vs. Mild Cognitive Impairment; MCI) and priming paradigm-related characteristics. This systematic review and meta-analysis provides a quantitative summary of repetition priming in AD. We examined the between-group standard mean difference comparing repetition priming in AD dementia or amnestic MCI (aMCI; presumably due to AD) to controls. Thirty-two studies were selected, including 590 individuals with AD dementia, 267 individuals with amnestic MCI, and 703 controls. Our results indicated that both individuals with aMCI and AD dementia perform worse on repetition priming tasks than cognitively older adults. Paradigm-related moderators suggested that the effect size between studies comparing the combined aMCI or AD dementia group to cognitively healthy older adults was the highest for paradigms that required participants to produce, rather than identify, primes during the test phase. Our results further suggested that priming in AD is impaired for both conceptual and perceptual priming tasks. Lastly, while our results suggested that priming in AD is impaired for priming tasks that require deep processing, we were unable to draw firm conclusions about whether priming is less impaired in aMCI or AD dementia for paradigms that require shallow processing.

**Supplementary Information:**

The online version contains supplementary material available at 10.1007/s11065-021-09504-5.

## Introduction

Repetition priming can be defined as the facilitation of item retrieval resulting from previous exposure to that item for which conscious recollection is not required (Schacter et al., 1987). In contrast, explicit (or declarative) memory, is the conscious recollection of facts and events (Squire, 2004). The dissociation of repetition priming from explicit memory was first shown by Warrington and Weizkrantz (Warrington & Weiskrantz, 1974), who demonstrated that individuals with “pure amnesia” did not consciously remember recently studied words. However, when presented with word-stems and asked to name the first words that came to mind to complete those word stems, these individuals were more likely to name the previously studied words than words that were not studied before. This dissociation of impaired explicit memory and intact priming prompted other researchers to aim to replicate these results.

Alzheimer’s disease (AD) is a neurodegenerative disease in which brain areas in the medial temporal lobe are typically affected most in its early stages, after which the pathology spreads to the posterior temporal lobes, parietal lobes, and frontal lobes (Sluimer et al., 2009; Whitwell, 2010). Explicit memory, which heavily relies on the medial temporal lobe (Eichenbaum & Lipton, 2008; van Strien et al., 2009), is often one of the first cognitive functions to be affected in AD, resulting in both retrograde and anterograde amnesia. As priming was initially shown to remain intact in amnestic individuals with medial temporal lobe damage, priming is typically considered to remain spared in the early stages of AD. However, while some studies have found spared priming in AD, many studies since then have suggested that priming does not remain intact in AD and that there are likely additional factors, such as test characteristics, that result in different findings across patient groups (Fleischman, 2007; Fleischman & Gabrieli, 1998; Millet et al., 2010; Schacter et al., 2004).

Many cross-sectional studies that aim to assess whether or not priming remains spared in AD do so by comparing priming performance in individuals with AD to cognitively healthy controls. If the standard mean difference between these two groups is small, this is taken as evidence that priming performance in individuals with AD is similar to the priming performance of healthy controls, which suggests that priming remains spared in AD. The most salient patient characteristic that may account for the heterogeneity in findings may be the differing levels of AD severity in patient samples within and across studies. Given the neurodegenerative nature of AD (Jack et al., 2018) cognitive function gradually declines over the course of the disease. The disease often first manifests as the Mild Cognitive Impairment syndrome (MCI), in which cognitive concern and objective cognitive impairment are present but instrumental activities of daily living remain largely intact (Albert et al., 2011). When declarative memory is impaired, these individuals are classified as having the amnestic subtype of MCI (or aMCI; Petersen, 2004). Individuals with aMCI often progress to a full AD dementia syndrome. As the neurodegeneration and consequent cortical atrophy in the dementia stage of AD are more profound than in the earlier stages of the disease, more consistent sparing of repetition priming would be expected when examining pre-stages of AD dementia such as aMCI. Similarly, after progression to AD dementia, one would expect better priming performance in the early or mild dementia phases than in the later moderate and severe dementia phases. However, the effect of AD severity on priming has not been examined thus far.

In addition to AD disease severity, there are several test characteristics that evoke different subtypes of priming and, as a result, different levels of complexity. Importantly, various task characteristics may rely on distinct cognitive processing operations dependent on different brain areas. This idea was previously outlined by Torres in 1994, who described that factors such as the type of stimuli in both the exposure and test phase, such as pictures or words, may be processed by different neural substrates (Torres & Raz, 1994). For instance, perceptual priming, typically picture priming, is thought to rely on the perceptual representation system, first described by Schacter (1990), which typically remains spared in early AD dementia (Golby et al., 2005; Keane et al., 1991; Kessels et al., 2011; Koivisto et al., 1996; Millet et al., 2008; Mitchell & Schmitt, 2006)*.* In contrast, conceptual or semantic priming is thought to rely on semantic memory, which is often affected in AD dementia (Brandt et al., 1988; Keane et al., 1991; Lazzara, 2001; Monti et al., 1996; Salmon et al., 1988; Vaidya et al., 1999). Despite this helpful distinction, inconsistent results are found even when this distinction is taken into account (Christensen & Birrell, 1991; Deason et al., 2012, 2015; Maki & Knopman, 1996; Martins & Lloyd-Jones, 2006; Perri et al., 2007; Vaidya et al., 1999). Perhaps these differences result from how perceptual and conceptual priming are defined. In the review of Fleischman and Gabrieli (1998), perceptual priming was defined as priming that relies on the visual as well as the auditory and tactual form of a target stimulus and that has minimal changes between the exposure and test phases. They argue that, as per these criteria, tasks such as degraded picture naming, word identification, and lexical decision tasks are largely perceptual in nature. Yet, considering the cognitive processes that are employed by such tasks, a different way to understand the difference between conceptual and perceptual priming is based on whether or not the *meaning* of the stimulus needs to be understood. For instance, if a task requires reading, letters are processed not just visually but also semantically.

Some priming tasks aim to induce a deeper level of processing during the exposure phase. A previous meta-analysis that compared word-stem completion priming performance in individuals with AD dementia to that of cognitively unimpaired older adults found that the standard mean difference between these groups varied based on what individuals were asked to do during the encoding phase (Millet et al., 2010). As described in this meta-analysis, participants are often asked to rate the likeability or pleasantness of the stimuli during the Word-Stem Completion task exposure phase (Millet et al., 2010). Other paradigms require participants to make a semantic judgement (e.g., “is this item larger than a shoebox”) or to provide a definition of the word during the encoding phase. Such evaluations are thought to be mediated by higher-level semantic encoding, requiring participants to think about the properties or meaning of the stimulus. Another encoding condition described in this meta-analysis was to ask participants to read the words in the encoding phase. While it is likely that participants semantically process words when reading, this cannot always be assumed with certainty. There was only one study included in this meta-analysis with an encoding phase that evoked shallow processing: the study of Carlesimo et al. (1999) required participants to count the vowels in the word stimuli. A last type of processing that has been utilized in the encoding phase involves having participants generate words themselves. In some studies, it is seen as a priming effect when these words are subsequently produced again in the test phase, (Fleischman et al., 1997, 1999; Grosse et al., 1990). One would expect the standard mean difference comparing priming between individuals with aMCI or AD dementia to cognitively healthy older adults to be larger for paradigms that require deep processing during the exposure phase than for paradigms that require shallow processing during the exposure phase.

Some additional challenges related to the level of processing during the exposure phase should be considered. For instance, if the task requires participants to produce the target words themselves, as described above, this can introduce a potential bias. For instance, in the study of Fleischman (Fleischman et al., 1997), participants were given a definition and asked to generate a word that matches that definition. In such a task, the target words are potentially standardized as there may only be one word that matches the definition. However, other studies used a paradigm that allows participants to freely generate words that will later serve as target words. For instance, the study of Grosse et al. (1990) had participants complete a set of sentences with single word endings. The single words that were generated by participants later served as primes. Thus, in such paradigms, the “primed words” are the first words that came to mind for the participants during the “exposure” or learning phase, which could mean that these words are, for instance, more frequent or more personally meaningful. This lack of standardization of target words is therefore problematic and paradigms that use suchs a target words likely do not rely on the same processes as paradigms using standardized target words.

The difference between identification and production repetition priming is another task-induced difference that might result in partially different cognitive processes being involved in priming (Gabrieli et al., 1999a, b). Identification priming tasks require the identification of a stimulus in the test phase, such as the identification of a fragmented picture. In production priming, participants are presented with a cue, which is meant to induce the production of a response. For instance, in the Word-Stem Completion Task, participants are presented with a word stem and are asked to generate a word that starts with that word stem. In production tasks, the target stimuli cannot be produced solely by identification of the cue. These tasks may place a larger demand on retrieval processes than identification priming tasks do, and therefore are more likely to be impaired in AD.

A major complication in the literature on repetition priming, also described in the narrative reviews of Fleischman and Gabrieli (1998) and Fleischman (2007), is the statistical power of the studies that have thus far been conducted. Specifically, many articles claiming that priming remains intact in AD have done so based on the absence of a statistically significant effect when comparing repetition priming between cognitively healthy older adults and individuals with AD. Concluding that performance is equivalent between groups based on the absence of a significant difference is, however, problematic. The absence of an effect can also be due to poor methodology or small sample sizes, which can result in insufficient power. Reporting between-group effect sizes can help gauge the magnitude of the effect in the absence of a statistically significant difference.

Besides the potential bias in the literature on repetition priming in AD that was introduced by studies drawing conclusions based on the absence of a statistically significant effect, several other factors may have contributed to inconsistencies among findings. As described above, these include several task-related differences such as how deeply stimuli are processed during the exposure phase, whether the task prompts conceptual or perceptual processing, and whether production or identification priming is required in the test phase. The integrative reviews of Fleischman (2007) and Fleischman and Gabrieli(1998) describe some of these factors in more detail. However, the integrative review format of these studies, as opposed to the meta-analytic format adopted in the present study, does not allow for the quantification of differences as a function of these factors.

Meta-analyses, as opposed to narrative review, offer the advantages of providing a synthesis of the results and a quantification of the differences. Previous meta-analyses have either used very narrow definitions of priming in AD, such as Millet and colleague’s review on word-stem completion priming (Millet et al., 2010) or have assessed the broader concept of implicit memory in AD (Meiran & Jelicic, 1995). These two earlier meta-analyses can thus be criticized with respect to the breadth of their focus. That is, if a meta-analysis aims to synthesize results that are too broad, the included studies can be too heterogenous. This issue of “comparing apples to oranges” can lead to a bias of the synthesized results and, thereby, misleading take-aways (Shelby & Vaske, 2008). In turn, using a definition that is too narrow may limit the potential added value of a meta-analysis as well (Shelby & Vaske, 2008).

The aim of the current meta-analyses was to address the issues of power, heterogeneity of disease severity, and heterogeneity of task characteristics when examining repetition priming. The issues of power were addressed by examining the standard mean difference of all studies that have compared repetition priming in AD dementia or aMCI to cognitively healthy older adults. For each individual study on this topic that allows for such a comparison, an effect size was calculated and added to the combined “meta” effect size based on all the individual studies. Issues of heterogeneity of disease severity and task characteristics were examined by moderator analyses. The current meta-analysis aimed to examine several moderators, namely 1) the AD disease severity stage, and several test characteristics such as 2) whether the paradigm induced conceptual versus perceptual priming, 3) the level of processing during the exposure phase, and 4) whether the paradigm required identification priming versus production priming during the test phase. In order to explore meaningful test characteristics while avoiding biased results from including studies that are too heterogeneous, only repetition priming studies were included and several relevant task characteristics were explored with the use of moderator analyses. Further, to avoid potential bias that would not lead to a more informative synthesis, studies that used priming paradigms with additional complexity that were not repeated by other studies will not be explored.

## Methods

### Systematic Search

For the literature search and study selection procedures the PRISMA guidelines were followed (Moher et al., 2009). A literature search of PsycInfo and MEDLINE databases was conducted by a librarian (NS) on 04/15/2020. The complete search strategy (which included PsycInfo and MeSH controlled vocabulary terms and keywords and language limits) can be found in Supplement 1. A flow diagram that delineates the numbers of the search results can be found in Supplement 2, Supplemental Fig. 1.

**Fig. 1:**
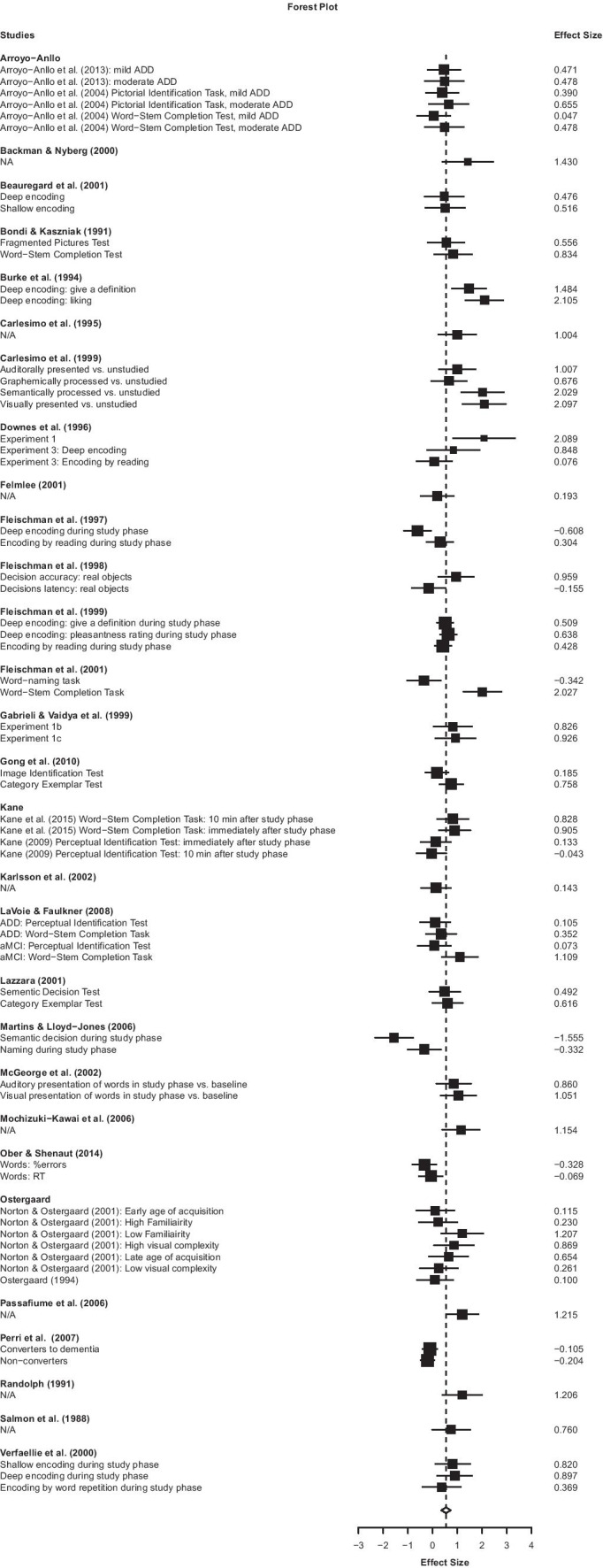
Forest plot depicting all the effect sizes included in the current meta-analysis, nested within study or study group. *Note*: Negative effect sizes indicate that there was more priming in aMCI or AD dementia patients than in healthy controls

#### Study Selection

The inclusion criteria were determined a priori and were assessed in the following order (1) the article was in English or Dutch (as authors LDW, RPCK, and VP are fluent in both languages), and (2) the study used a minimum of one implicit learning or memory task. The memory or learning task was completed by (3) a group of individuals diagnosed with amnestic Mild Cognitive Impairment or AD dementia and (4) a control group of cognitively healthy older adults. In addition, (5) the article was peer reviewed (dissertations were considered to be peer reviewed, because most committees consist of at least two experts on the topic) and (6) the article was original work (hence, review articles were excluded). The topic of the current meta-analysis was narrowed down further by two additional criteria: (7) the article included a repetition priming task that was completed by patient and control groups and (8) the statistics allowed a statistical comparison of repetition priming between the patient and control group. The specifics regarding the statistics that were needed are described in the “Data Synthesis” section below. Authors LDW and DOS reviewed the list of potential articles independently.

For repetition priming tasks, we only considered studies that repeated the same stimulus concepts in the exposure and test phases. Priming paradigms that used related but different stimuli in the exposure and test phases (such as the concept “cat” in the exposure phase to prime the item “dog” in the test phase) were excluded. However, the exposure and test item did not necessarily have to occur in the same modality (e.g., if participants first read the word “dog” and had to identify a fragmented picture of a dog during the testing phase, this was considered repetition priming). Typical repetition priming paradigms have an exposure either immediately followed by the testing phase or with a short delay. However, there were five studies that had a testing phase a year or more after the exposure phase (Beatty et al., 1998; Carlesimo et al., 1998; Mochizuki-Kawai et al., 2006; Ostergaard, 1994; Perri et al., 2007). A long delay is expected to yield different results when compared to same-day priming experiments, particularly given that individuals with aMCI or AD dementia often decline over time. Therefore, these results were omitted in the current meta-analysis. This resulted in the exclusion of one study that only reported longitudinal priming effects (Beatty et al., 1998). For the other four studies, the short-term priming results were included in the current meta-analysis. Additionally, we were interested in priming as an alternative long-term memory system. Declarative long-term memory is often assessed by (repeated) exposure to stimuli, after which the ability to remember the items is tested, often both directly after the learning phase as well as 20–30 min after the learning phase. We therefore excluded “short-term priming” paradigms that did not have separate exposure and test phases but instead had trials consisting of the exposure to a stimulus immediately followed by testing for that specific stimulus, as these paradigms resemble “priming working memory” more than long-term memory. To avoid too much heterogeneity in the type of priming paradigm, we further excluded results from studies that only used priming paradigms with additional complexity. For instance, priming paradigms that primed at a sentence level instead of an item level, emotion-affective priming (e.g. LaBar et al., 2005), or priming with distractors to assess interference (Hogge et al., 2008), as these may not reflect the same process.

#### Overlapping Studies or Study Samples

The results reported in two dissertations were later published in peer reviewed journals. In these specific instances, the published results were used rather than the dissertations themselves, which were excluded to avoid repeated results within the meta-analytic model (Kane et al., 2015; Norton & Ostergaard, 2001), unless results on an additional task was reported in the dissertation but not in the published manuscript, in which case the results of the task that was not published were included from the dissertation (Kane, 2009). For studies that appeared to be from the same research group, authors were contacted to assess whether the study samples of these studies had overlap. We received one email that stated that there was no overlap between study samples (Fleischman et al., 1997, 1998, 2001; Gabrieli et al., 1999a). Other authors did not reply. For these studies, articles from the same research groups were nested. The article for which this was done is depicted in the Forest Plot in Fig. 1.

### Data Synthesis

#### Data Extraction

Authors BJ, PT, and LDW extracted data from reading full texts. To ensure accuracy all statistics were independently reviewed by two of these three data extractors. VP or MM was consulted in case of doubt on which statistics to pull for the effect size calculation. The statistics used for the calculations are reported in Table 1 and the calculated effect sizes are reported in the Forest Plot in Fig. 1. Demographic information on the participant groups, including the *n*, age, percentage of male versus female participants, and the diagnostic criteria used for the patient groups can be found in Table 2. When the exposure phase was repeated multiple times, only the effect size after one exposure phase was reported (Ostergaard, 1994). Further, there was one study for which the priming task included both words and non-words (Ober & Shenaut, 2014). However, for the non-words, no priming effect was found and therefore only the priming results for the words were used in the current meta-analysis. Similarly, a study that tested priming on real objects versus pseudo-objects did not find a priming effect for pseudo-objects. Therefore only the priming results for the real objects were used in the current meta-analysis (Fleischman et al., 1998).

**Table 1:** Tasks and outcome measures of the included studies

Authors	Year	Task	Outcome	ES calculated from:
aMCI				
Gong et al.	(2010)	Image Identification Test; Category Exemplar Test	Naming correct responses	*M*_*PrimedPooled*_ *SD*_*PrimedPooled*,_ *N*
LaVoie and Faulkner	(2008)	Word-Stem Completion Test; Perceptual Identification Test	Difference in percentage of stems completed with primed vs. non-primed words	*M*_*PrimedPooled*_ *SD*_*PrimedPooled*,_ *N*
Perri et al.	(2007)	Word-Stem Completion Test	Difference between number of stems completed with primed vs. non-primed words	*M*_*PrimedPooled*_ *SD*_*PrimedPooled*,_ *N*
AD Dementia				
Arroyo-Anllo et al.	(2013)	Lexical Priming Task	Difference between number of stems completed with primed vs. non-primed words	*M*_*PrimedPooled*_ *SD*_*PrimedPooled*,_ *N*
Arroyo-Anllo et al.	(2004)	Pictorial identification task	Difference between number of stems completed with primed vs. non-primed stimuli	*M*_*PrimedPooled*_ *SD*_*Unprjmed,*_* N*
Backman and Nyberg	(2000)	Word-Stem Completion Test	Difference between number of stems completed with primed vs. non-primed words	*F, N*
Beauregard et al.	(2001)	Semantic Judgment Task	Difference in percentage of stems completed with primed vs. non-primed words	*M*_*PrimedPooled*_ *SD*_*Unprimed,*_* N*
Bondi and Kaszniak	(1991)	Word-Stem Completion Test; Fragmented Pictures Test	Difference in percentage of stems completed with primed vs. non-primed words	*t, N; M*_*PrimedPooled*_ *SD*_*Unprimed,*_* N*
Burke et al. Different study phases aggregated	(1994)	Word-Stem Completion Test	Difference between number of stems completed with primed vs. non-primed words	*M*_*PrimedPooled*_ *SD*_*Unprimed*,_ *N*
Carlesimo et al.	(1999)	Word-Stem Completion Test	Difference in percentage of stems completed with primed vs. non-primed words	*M*_*PrimedPooled*_ *SD*_*Unprimed,*_* N*
Carlesimo et al.	(1995)	Word-Stem Completion Test	Difference between number of stems completed with primed vs. non-primed words	*t, N*
Downes et al.	(1996)	Word-Stem Completion Test	The number of target (primed) completions divided by the total number of completions	*M*_*PrimedPooled*_ *SD*_*Unprimed,*_* N*
Felmlee	(2001)	Degraded-Figures Priming Task	Proportion of fragmented images correctly identified for primed and non-primed pictures	*M*_*PrimedPooled*_ *SD*_*Unprimed,*_* N*
Fleischman et al.	(2001)	Word naming task and Word-Stem Completion Test	Ms Percentage correct response	*M*_*PrimedPooled*_ SD_Unprimed_*;* *F, N*
Fleischman et al.	(1999)	Word-Stem Completion Test	Difference between number of stems completed with primed vs. non-primed words	*M*_*PrimedPooled*_ *SD*_*PrimedPooled*,_ *N*
Fleischman et al.	(1998)	Object Decision Making Task	Difference in latency mean of median RT and percentage correct in primed vs. non-primed items	*M*_*PrimedPooled*_ *SD*_*Unprjmed,*_* N*
Fleischman et al. Different study phases aggregated	(1997)	Word-Stem Completion Test	Difference between number of stems completed with primed vs. non-primed words	*M*_*PrimedPooled*_ *SD*_*Unprjmed,*_* N*
Gabrieli et al.	(1999)	Word-Stem Completion Test primed with pictures(1b); Word-Stem completion (1c)	Completing stems to form study-list words more often than by chance	*F, N* *F, N*
Kane et al.	(2015)	Word-Stem Completion Test	Difference between number of stems completed with primed vs. non-primed words	*F, N*
Kane Experiment 1	(2009)	Perceptual Identification Task	Mean identification time (in ms)	*M*_*PrimedPooled*_ *SD*_*Unprjmed,*_* N*
Karlsson et al.	(2002)	Visual word fragment completion	Difference in magnitude of proportions between primed and unprimed fragments	*M*_*PrimedPooled*_ *SD*_*PrimedPooled,*_* N*
Lazzara	(2001)	Semantic decision task; Category exemplar generation task	Difference in RT for old and new stimuli divided by the new stimuli RT; Difference in target items between primed and non-primed stimuli	*F, N; M*_*PrimedPooled*_ *SD*_*PrimedPooled,*_* N* *M*_*PrimedPooled*_ *SD*_*Unprimed,*_* N*
Martins and Lloyd-Jones	(2006)	Category Exemplar task	Mean percentage correct responses	*M*_*PrimedPooled*_ *SD*_*Unprimed,*_* N*
McGeorge et al.	(2002)	Word-Stem Completion Test	Difference in mean proportion of items correctly recalled between primed and non-primed words	*M*_*PrimedPooled*_ SD_Unprimed,_
Mochizuki-Kawai et al.	(2006)	Picture-Fragment Completion Task	Identification threshold between primed and non-primed pictures	*M*_*PrimedPooled*_ *SD*_*Unprimed,*_* N*
Norton and Ostergaard	(2001)	Picture fragment naming task	Difference in the level of fragmentation at which items were correctly named between primed and non-primed stimuli	*M*_*PrimedPooled*_ SD_Unprjmed,_ *N*
Ober and Shenaut	(2014)	Lexical Decision Making Task	RT; percentage errors for primed vs. non-primed stimuli	*M*_*PrimedPooled*_ *SD*_*Unprjmed,*_* N*
Ostergaard	(1994)	word-identification (naming) task	Mean identification threshold new vs previously exposed words	*M*_*PrimedPooled*_ *SD*_*Unprimed,*_* N*
Passafiume et al.	(2006)	Word-Stem Completion Test	Percentage of correct response (i.e. stems completed with the expected words) transformed in arcsine	*F, N*
Randolph	(1991)	Degraded-Figures Priming Task	Difference between number of stems completed with primed vs. non-primed words	*t, N*
Salmon et al.	(1988)	Word-Stem Completion Test	Tendency to produce the second word of the related word pairs	*t, N*
Verfaellie et al.	(2000)	Auditory priming task	Difference in ratio of correct identification between primed and non-primed stimuli	*M*_*PrimedPooled*_ *SD*_*Unprjmed,*_* N*

*AD* Alzheimer’s Disease,*FF*-statistic of a two by two interaction effect between familiar vs. unfamiliar condition and patient group vs. HC, *M* mean, *SD* Standard Deviation, *SD*_priming_ Standard Deviation of the priming effect, *M*_priming_ mean of the priming score, *SE* Standard Error, *SE*_*primed*_ Standard Error of the primed stimuli/condition, *SE*_unprimed_ Standard Error of the stimuli that were not primed, *V* Variance of Hedges *g*

**Table 2:** Demographic characteristics of the study samples

Authors	Year	Patient Group & Diagnostic Criteria	HC n	MCI/ ADD n	HC mean age	MCI/ADD mean age	HC %male	MCI/ ADD%male
aMCI								
Gong et al.	(2010)	ADD; NINCDS-ADRDA	35	35	69.1	68.7	54.3	48.6
LaVoie and Faulkner	(2008)	aMCI: Petersen (subjective memory complaint, objective memory dysfunction: LM WMS-III = 1–1.5 SD < norms, CDR 0.5, MMSE score >24; normal ADL); ADD: NINCDS-ADRDA	26	26 aMCI; 16 ADD	68.85	aMCI: 71.54; ADD: 15.30	38.5	aMCI: 53.8; AD: 50.0
Perri et al. Dementia non-converters	(2007)	ADD; NINCDS-ADRDA	87	111	68.0	67.8	41.4	42.3
Perri et al. Dementia converters	(2007)	ADD; NINCDS-ADRDA	87	79	68.0	73.2	41.4	44.3
AD Dementia								
Arroyo-Anllo et al.	(2013)	ADD; NINCDS-ADRDA	15	Mild ADD: 20; Mod: 10	78.1	Mild ADD:79.2Moderate:82.3	NR	NR
Arroyo-Anllo et al.	(2004)	ADD; NINCDS-ADRDA	15	Mild ADD: 20; Mod: 10	78.1	Mild ADD:79.2Moderate:82.0	NR	NR
Backman et al.	(2000)	ADD; NINCDS-ADRDA	9	8	62.7	60.2	44.4	37.5
Beauregard et al.	(2001)	Petersen, 2004; CDR 0-.5; no ADL deficits	12	11	74.7	77	41.7`	36.4
Bondi and Kaszniak	(1991)	ADD; NINCDS-ADRDA	16	12	69.6	70.7	43.8	66.7
Burke et al.	(1994)	ADD; NINCDS-ADRDA	20	20	78.1	79.4	30	35
Carlesimo et al. experiment 1	(1999)	ADD; NINCDS-ADRDA	15	15	66.1	67.1	50	50
Carlesimo et al.	(1995)	ADD; NINCDS-ADRDA	18	11	66.5	63.8	27.8	54.6
Downes et al. experiment 1, experiment 3 (both “liking” and reading conditions)	(1996)	ADD; NINCDS-ADRDA	7 14	7 14	66.1 67.8	67.6 67.5	NR	NR
Felmlee	(2001)	NINCSD/ADRDA	16	16	74.1	81.5	NR	NR
Fleischman et al.	(2001)	NIAA + AD Etiology	16	16	70.4	73.4	NR	NR
Fleischman et al.	(1999)	ADD; NINCDS-ADRDA	58	91	75.8	75.6	NR	NR
Fleischman et al.	(1998)	ADD; NINCDS-ADRDA	16	16	70.4	73.4	NR	
Fleischman et al.	(1997)	ADD; NINCDS-ADRDA	24	28	71.5	72.8	29.5	22.2
Gabrieli et al. Exp. 1b Exp 1c	(1999)	ADD; NINCDS-ADRDA	14 12	12 12	67.3 67.9	71.8	16.7 28.6	50.0
Kane et al.	(2015)	ADD; NINCDS-ADRDA	20	20	87.7	80.2	45	65
Kane	(2009)	ADD; NINCDS-ADRDA	20	20	87.7	80.2	42.0	53.0
Karlsson et al.	(2002)	ADD; NINCDS-ADRDA	20	20	64.1	67.1	40	40
Lazzara	(2001)	ADD; NINCDS-ADRDA	19	20	75.2	77.1	26.3	40
Martins and Lloyd-Jones	(2006)	ADD; NINCDS-ADRDA	16	16	73.5	75.6	43.8	56.3
McGeorge et al.	(2002)	ADD; NINCDS-ADRDA	16	16	68.8	70.5	NR	NR
Mochizuki-Kawai et al.	(2006)	ADD; NINCDS-ADRDA	17	13	67.2	72.5	41.2	38.5
Norton and Ostergaard	(2001)	ADD; NINCDS-ADRDA	12	12	75.3	75.2	NR	NR
Ober and Shenaut	(2014)	ADD; NINCDS-ADRDA	34	30	71.5	78.4	NR	NR
Ostergaard	(1994)	ADD; NINCDS-ADRDA	15	12	63.3	68.5	66.7	58.3
Passafiume et al.	(2006)	ADD; NINCDS-ADRDA	20	23	68.3	70.2	50	43.5
Randolph	(1991)	ADD; NINCDS-ADRDA	20	10	77.7	75.2	NR	NR
Salmon et al.	(1988)	ADD; NINCDS-ADRDA	13	13	66.5	71.2	61.5	23
Verfaellie et al.	(2000)	ADD; NINCDS-ADRDA	16	16	72.5	75.4	62.5	87.5

*AD* Alzheimer’s disease, *ADD* Alzheimer’s Disease Dementia, *HC* Healthy Controls, *MCI* Mild Cognitive Impairment, *NR* Not reported, *NINCDS-ADRDA* National Institute of Neurological and Communicative Diseases and Stroke/Alzheimer's Disease and Related Disorders Association

#### Repetition Priming Statistics and Effect Size Calculation

All outcome measures that were included are provided in Table 1. To gauge the reliability of the priming effects across test characteristics, priming *Z-*scores were calculated by dividing the priming mean by the priming *SD* separately for the control and patient groups*.* For studies that did not report within-group priming scores but reported the mean and *SD* (or *SE*) of the performance on both unprimed stimuli and primed stimuli, within-group mean priming scores were calculated as follows: *Z*_*Primed*_ = ($M_{Unprimed} - M_{Primed}$) / $S{D}_{Unprimed}$. Instead of the pooled *SDs* or *SEs*, the *SDs* or *SEs* of the non-primed stimuli were used in these studies (producing a maximally conservative estimate).

The first author calculated the between-group effect sizes (Cohen’s *d*s*)*, which were then converted to Hedges’ *g* to correct for the bias that can be introduced when having small sample sizes. These calculations were based on the *n* for the patient and control groups, along with one of the following sets of statistics:

Statistics on priming scores. These statistics were calculated by subtracting the performance after the exposure or learning phase from the performance before exposure or learning phase (or on stimuli that participants were not previously exposed to). For studies that reported the priming scores of each group together with the standard deviation (*SD;* or standard error of the mean: *SEM*) of the priming score, Cohen’s *d* between groups were calculated using the following standardized methods:

$d=({M}_{PrimedControls}-{M}_{PrimingAD})/{SD}_{PrimedPooled}$ In addition, *SD*_*PrimedPooled*_ was calculated as follows:

$$SDPrimedPooled=\sqrt{\frac{\textit{SD}_\textit{PrimedControls}^2\left({\textit{n}}_\textit{PrimedControls }-1\right)\text{ + }\textit{SD}_\textit{PrimedAD}^2({\textit{n}}_\textit{PrimedAD}{-1}}{{\textit{n}}_\textit{Controls}+{\textit{n}}_\textit{AD}{ - }2}}$$

When a *SEM* was reported instead of a *SD*, *SEM* was converted to *SD*. For studies that did not report within-group priming scores but reported the mean and *SD* (or *SE*) of the performance on both non-primed stimuli and primed stimuli, within-group mean priming scores were calculated as follows: *M*_*PrimedPooled*_ = $M_{Unprimed} - M_{Primed}$. Cohen’s *d* was calculated as described above for these within-group priming scores. Instead of the pooled *SDs* or *SEs*, the *SDs* or *SEs* of the non-primed stimuli were used in these studies (producing a maximally conservative estimates of effect size).

1. For studies that analyzed a two-by-two interaction effect on condition (primed versus non-primed by patient group versus control group), we used the *F-*statistic of the interaction effect to calculate Cohen’s *d* as follows: $d = \sqrt {F(n_{Controls} + n_{AD} ) \times (n_{Controls} \times n_{AD} )}$.

#### Hedges’ g

The Practical Meta-Analysis Effect Size Calculator by Wilson was used to derive separate between-group effect sizes (Cohen’s *d*) and effect size variances (retrieved in March 2019; Wilson, n.d.). Because Cohen’s *d* can be inflated in small sample sizes, we converted Cohen’s *d* to Hedges’ *g* (Borenstein et al., 2009). Hedges’ *g* was derived as follows: $g = \frac{d \times 1 - 3}{{4 \times (df - 1)}}$. The variance of Hedges’ *g* was calculated based on correction factor *J*. *J* is often calculated as follows: $J = 1 - (\frac{3}{4 \times df - 1})$, in which *df* refers to the residual degrees of freedom. In our meta-analysis, a negative Hedges’ *g* indicates that there was more repetition priming in individuals with aMCI or AD than in cognitively unimpaired controls.

### Analyses

#### Analytic Model

The current meta-analysis employed a random-effect model. Random effect models are preferred when heterogeneity is expected between studies based on variables such as the outcome measures, type of participants, or type of task (Borenstein et al., 2009). The Robust Variance Estimation (RVE) method function for linear mixed effect models was used (Fisher & Tipton, 2015) in R software (Team, 2016). This recently developed method was built as a new approach to handle the issue of non-independent effect sizes that often bias meta-analyses when, for instance, multiple dependent variables are included per study or sample sizes appear to overlap. This allows researchers to avoid giving more weight to studies that list more than one *DV*, participant groups of interest, study or test conditions, or priming tasks in the calculation of the meta-effect size. Other advantages of this package include a correction for skewed covariances between studies and a correction for small sample sizes, which is recommended for meta-analyses with less than 40 included studies (Fisher & Tipton, 2015). The RVE function requires the input of the effect sizes of each individual study and the variance of each individual effect size. Table 2 shows which dependent variables (*DV*s*)*, patient groups of interest, study or test conditions, and priming tasks were included per study. The R-code can be found in Supplement 3.

#### Subgroup Analyses

In random-effects models, the differences in results across studies are assumed to be due to measurable differences reflected in variation between studies that is greater than would be expected if the true effect were the same in all studies. This variability is considered to be “true heterogeneity.” We report the τ^*2*^ statistic, which indicates the between study variance (Deeks et al., 2019) and distinguished “true heterogeneity” from random error using the *Q* statistic, a measure of weighted squared deviations (Borenstein et al., 2009). When true heterogeneity is present, it can be further explored by subgroup or moderator analyses.

In the current sub-analyses, we assessed four binary moderators. The first moderator was AD disease stage (MCI versus dementia). We examined if the overall effect size (Hedges’ *g*) for all studies that compared individuals with aMCI to cognitively healthy older adults was smaller than the Hedges’ *g* for all studies that compared individuals with AD dementia to healthy controls.

**Fig. 2:**
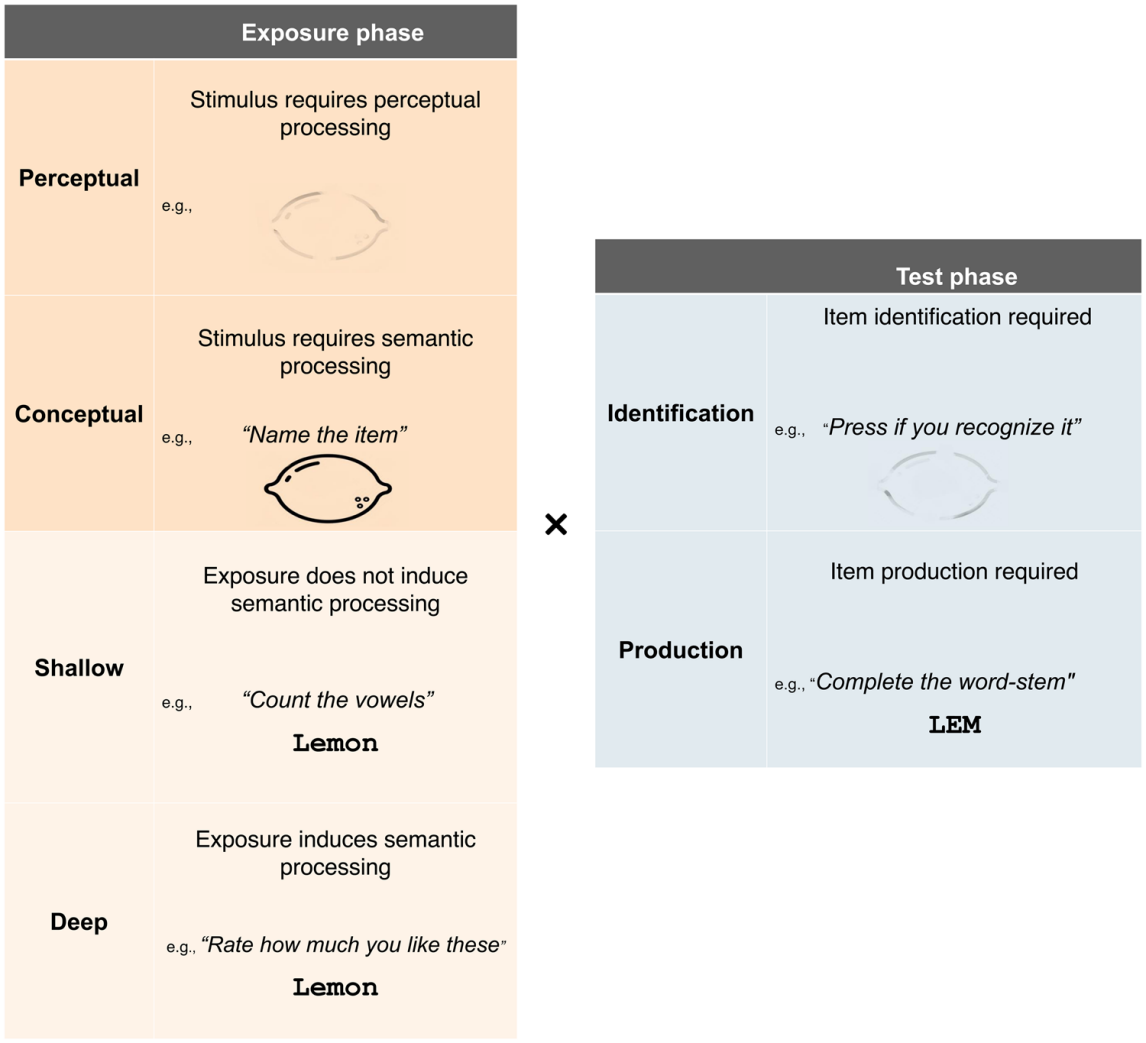
A schematic view of examples of the repetition priming characteristics that are tested in the moderator analyses

The second to fourth moderators were all related to the priming task paradigm characteristics and examples of these task characteristics are depicted in Fig. 2. The second moderator was conceptual versus perceptual priming. Priming was only considered perceptual when both the exposure and test phase were visual in nature and repeated the same stimulus in the test and exposure phases.

The third moderator was the level of processing during the exposure phase. We compared test paradigms that required deep processing to paradigms that required shallow processing during the exposure phase. Deep processing was defined as any type of processing that required participants to semantically process the items. For instance, “rate these items with regards to how much you like them” or “say yes if these items are larger than a shoe box, say no if they are smaller.” Shallow processing was defined as processing that does not require any understanding of the meaning of the stimulus, for instance, “count the vowels of each word.”

The fourth moderator was the level of processing during the test phase. Specifically, we assessed the difference between test paradigms that required participants to recognize items during the testing phase (e.g., identification of an object in a fragmented picture task) and test paradigms that required participants to (re)produce items during the testing phase (e.g. the production of words in the Word-Stem Completion Task). We expected that the Hedges’ *g* comparing priming between individuals with aMCI or AD dementia to cognitively healthy older adults would be larger for paradigms that required participants to produce answers than for paradigms that required the identification of stimuli during the test phase.

#### Publication Bias

Publication bias often results from the phenomenon that studies without statistically significant findings get published less frequently. When this occurs, it can bias meta-analysis results (Borenstein et al., 2009). Publication bias was assessed in several ways in the current meta-analysis: 1) We included dissertations in the current meta-analyses. The inclusion of dissertations or other forms of non-published data can lower publication bias. To assess for a bias, dissertations and journal articles were ran as a binary moderator (unpublished vs. published) in an additional analysis to compare whether there are systematic differences between these two categories, 2) We depicted the relationship between the individual effect sizes of the studies that are included in the meta-analysis and their standard error by the use of a funnel plot. In a funnel plot, studies that have a smaller standard error, typically larger studies, often cluster around the mean effect size. Studies with a larger standard error, typically smaller studies, are displayed towards the bottom of the graph. Smaller studies often have more sampling error variation in effect sizes and their effect sizes tend to be more spread out. These relationships are shown in the “funnel” shape of a funnel plot (Borenstein et al., 2009); 3) We used the Rank Correlation Test for Funnel Plot Asymmetry to assess the significant asymmetry of the effect sizes and their variance for the included studies. The latter two methods to assess for publication bias were ran using the Metafor package (Viechtbauer, 2010) in R (Team, 2016).

## Results

### Included Studies

The final study sample of the current meta-analysis consisted of 32 articles (*k* = 32; *N* = 1,422). Three studies included an aMCI patient group and 30 studies included an AD dementia patient group. One study included both patient groups, namely, LaVoie and Faulkner (2008). Summing up the number of participants in all studies, the total number of individuals with AD dementia that completed the tasks was 590, the total number of individuals with amnestic MCI that completed the tasks in these studies was 267, and the total number of healthy older adults that completed the tasks in these studies was 703. Table 2 delineates the sample size for each individual study.

### Priming Effect

To gauge the reliability of the priming effect across test characteristics, the mean, *SD*, minimum and maximum of the priming *Z-*scores (priming Mean divided by priming *SD*) are listed in Table 3, separately for the control and patient groups and across test characteristics*.* As can be seen in Table 3, the priming Z-scores were quite similar across the moderator. Further, authors of all articles that did not report statistics that allowed for the calculation of *Z*-scores reported that a priming effect was present for both (Bäckman et al., 1997; Carlesimo et al., 1995; Gabrieli et al., 1999a, b; Kane et al., 2015; Lazzara et al., 2001; Passafiume et al., 2006; Randolph, 1991; Salmon et al., 1988) or one (Bondi & Kaszniak, 1991) of the groups.

**Table 3:** Priming Z-scores across task characteristics

		Priming Z-scores			
		Mean	SD	Min	Max
AD/aMCI	Conceptual	1.413	1.053	-0.167	4.796
	Perceptual	0.842	1.091	-0.538	4.180
	Shallow	1.138	1.022	0.165	3.727
	Deep	1.301	1.174	-0.538	4.796
	Identification	1.030	1.299	0.047	4.796
	Production	1.399	0.956	-0.538	3.934
Controls	Conceptual	1.819	1.167	0.000	6.547
	Perceptual	1.442	1.062	0.236	4.300
	Shallow	1.376	1.098	0.000	3.085
	Deep	1.836	1.236	0.236	6.547
	Identification	1.279	1.029	0.000	4.300
	Production	1.946	1.147	0.236	6.547

*AD*  Alzheimer’s Disease dementia, *aMCI* amnestic Mild Cognitive Impairment, *SD* Standard Deviation of the Priming Z-scores, *Min.* Minimum of the Priming Z-scores, *Max*. maximum of the Priming Z-scores

### Meta-Analysis Effects

All the effect sizes calculated as part of the current meta-analysis, including the 29 study clusters and 68 individual effect sizes, are listed and depicted in the forest plot in Fig. 1. The meta Hedges’ *g* size was 0.547 (*SE* = 0.093, 95% *CI* [0.354,0.740], τ^2^ = 0.142). There was a statistically significant difference in repetition priming between individuals with aMCI or AD dementia and cognitively healthy older adults (*t*(20.4) = 5.900, *p* < 0.001).

### Subgroup Analyses

Sub-analyses were conducted to assess if the amount of *true variation* between studies could be explained by binary moderators. The first moderator assessed was AD disease stage (MCI versus dementia). The Hedges’ *g* between studies comparing individuals with aMCI to cognitively healthy older adults versus those comparing individuals with AD dementia to healthy older adults was -0.321 (*SE* = 0.269, 95% *CI* [-1.300, 0.658], τ^2^ = 0.122), indicating that the Hedges’ *g* between studies comparing repetition priming individuals with aMCI to cognitively healthy older adults was not significantly lower than the Hedges’ *g* comparing individuals with AD dementia to cognitively healthy controls (*t*(2.43) = -1.190, *p* = 0.336). The intercept reflected the studies comparing individuals with aMCI to cognitively healthy older adults Hedges’ *g* = 0.258, *SE* = 0.244, 95% *CI* [-0.816,1.330], *t*(2.43) = -1.19, *p* = 0. 0.336, and showed that there was no significant difference in priming between individuals with aMCI and cognitively healthy older adults. The second moderator assessed was the perceptual versus conceptual level of priming. The Hedges’ *g* comparing priming between our groups of interest (individuals with aMCI or AD dementia to cognitively healthy controls) using conceptual priming paradigms was 0.115 (*SE* = 0.137, 95% *CI* [-0.247, 0.478], τ^2^ = 0.167). The Hedges’ *g* between studies comparing aMCI or AD dementia to cognitively healthy older adults was not statistically higher in conceptual priming paradigms than in perceptual priming paradigms (*t*(6.21) = 0.976, *p* = 0.442). The intercept reflected the difference between individuals with aMCI or AD dementia versus controls for perceptual priming tests and was significant (Hedges’ *g* = 0.454, *SE* = 0.067, 95% *CI* [-0.248,0.660], *t*(3.1593) = 6.833, *p* = 0.005). Thus, there was a significant difference in repetition priming between individuals with MCI or AD versus cognitively healthy older adults on perceptual priming tasks.

The third moderator assessed was the level of processing during the exposure phase (shallow versus deep processing). The Hedges’ *g* comparing priming between our groups of interest (individuals with aMCI or AD dementia versus cognitively healthy controls) using paradigms that required deep processing during the exposure phase versus paradigms that required only shallow processing was -0.236 (*SE* = 0.218, 95% *CI* [ -0.722, 0.251], τ^2^ = 0.201); the Hedges’ g between studies comparing aMCI or AD dementia to cognitively healthy older adults was not significantly higher in paradigms that required deep processing than in paradigms that required shallow processing (*t*(9.780) = -1.080, *p* = 0.305). The intercept reflected the difference between individuals with aMCI or AD dementia versus controls for conceptual priming tests and was significant (Hedges’ *g* = 0.616, *SE* = 0.122, 95% *CI* [0.356, 0.876], *t*(15.13) = 5.04, *p* < 0.001).

The fourth moderator assessed was the level of processing during the test phase (production versus identification). The Hedges’ *g* comparing priming between our groups of interest (individuals with aMCI or AD dementia versus cognitively healthy controls) using paradigms that required production priming versus paradigms that required identification priming was 0.473 (*SE* = 0.202, 95% *CI* [-0.016, 0.929], τ^2^ = 0.167). Hedges’ *g* was higher in paradigms that required production priming than for paradigms that required identification priming. This moderator was statistically significant (*t*(8.960) = 2.340, *p* = 0.044). The intercept reflected the difference between individuals with aMCI or AD dementia versus controls for studies that required identification during the test phase and was not statistically significant (Hedges’ *g* = 0.205, *SE* = 0.168, 95% *CI* [-0.0222,0.632], *t*(5.180) = 1.220, *p* = 0. 274).

### Publication Bias

**Fig. 3:**
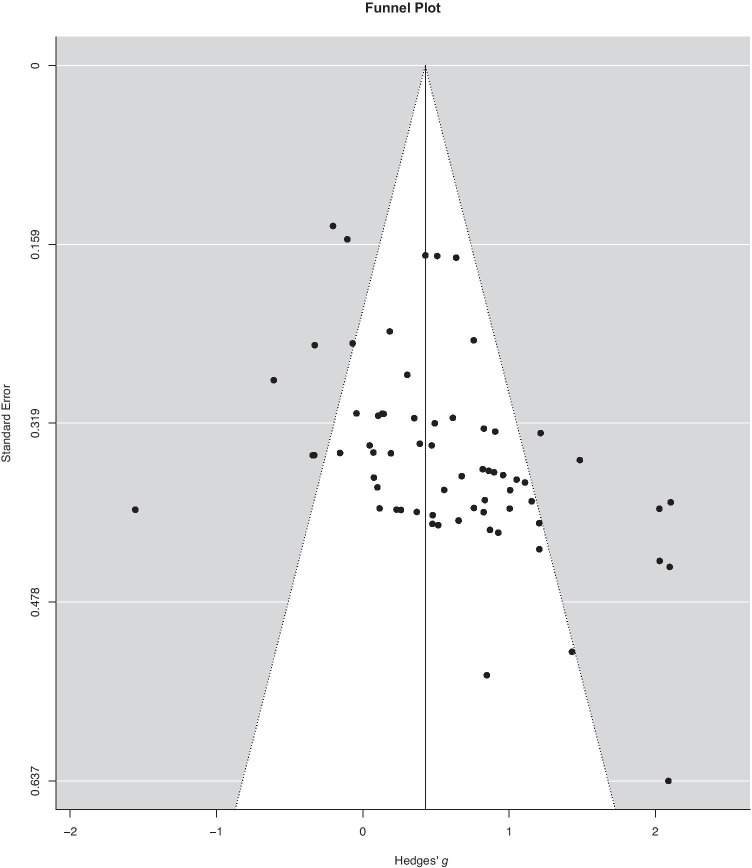
Funnel Plot showing the relationship between the individual Hedges’ *g*s and their standard error

The binary moderator (unpublished vs. published) to compare whether there are systematic differences between dissertations and journal articles was not statistically significant (*t*(4.100) = -1.310, *p* = 0.259). However, the Funnel Plot, shown in Fig. 3, demonstrates that higher effect sizes tended to have a higher standard error. The Rank Correlation Test for Funnel Plot Asymmetry was statistically significant (Kendall's τ = 0.342, *p* < 0.001), indicating that there was statistically significant asymmetry in the Hedges’ *g* and the variance for the included studies. Specifically, studies with higher standard mean differences had higher standard errors, suggesting that the overall standard mean difference could be inflated. This inflation could mean that the true difference in priming between individuals with aMCI or AD dementia and health older adults may be smaller than the differences reported in the meta-analysis above.

## Discussion

### Systematic Review Findings

The current systematic review and meta-analysis, consisting of 32 included articles, provides a quantitative summary of repetition priming in AD. The analysis examined the Hedges’ *g* of repetition priming in AD dementia or aMCI, presumably due to AD, compared to cognitively healthy older adults. This meta-analysis included three studies with an aMCI patient group and 30 studies with an AD dementia patient group, yielding a total of 590 individuals with AD dementia, 267 individuals with aMCI, and 703 cognitively healthy older adults that completed the priming tasks. The breadth of our literature review allowed us to examine important moderators including AD disease severity (i.e., amnestic MCI versus AD dementia stage), and paradigm-related factors including the type of priming (conceptual versus perceptual), the level of processing during the exposure phase (shallow versus deep processing), and the level of processing during the test phase (production versus identification).

### Meta-Analysis Findings

Regarding the level of AD severity, we found a statistically significant difference in repetition priming between individuals with aMCI or AD dementia versus cognitively healthy older adults, with the latter group showing more priming than the patient groups, in line with earlier reviews (Fleischman, 2007; Fleischman & Gabrieli, 1998; Meiran & Jelicic, 1995). Sub-analyses showed that there was no statistically significant difference in repetition priming when comparing individuals with aMCI or a combined aMCI or AD dementia group to cognitively healthy older adults, but that priming was significantly impaired in both the aMCI and AD dementia patient groups, suggesting that the neurodegeneration and consequent cortical atrophy already starts to impact brain areas that are important for repetition priming in the MCI stage of the disease.

In addition to AD disease severity, there are several paradigm-related characteristics that evoke different subtypes of priming and, as a result, different levels of complexity. When examining paradigm-related moderators, we found the that the Hedges’ *g* between studies comparing aMCI or AD dementia to cognitively healthy older adults was highest (indicating larger differences in priming between the patient and control groups) for paradigms that required participants to *produce* rather than *identify* primes during the test phase. Our results are consistent with the reviews of Fleischman and colleagues (Fleischman, 2007; Fleischman & Gabrieli, 1998). Our results confirm earlier suggestions that production versus identification during the test phase is an important predictor of performance, with priming being more impaired in aMCI and AD dementia for tasks that require production priming (Fleischman, 2007). As with explicit memory, the priming proces can be divided into the encoding, storage, and retrieval stage and the difference in production and identification priming may reflect difficulty with the retrieval stage, specifically. While the medial temporal lobe is known to be vital for long-term declarative memory, the prefrontal cortex is also known to be crucial for both encoding and retrieval (Simons & Spiers, 2003). Similarly, it may be that retrieval in priming also relies on prefrontal processes. While AD pathology is thought to first impact the medial temporal lobe, it is known to spread to the prefrontal cortex as the disease progresses (Sluimer et al., 2009; Whitwell, 2010), which may explain the more pronounced deficit in production versus identification priming in AD. While this hypothesis has not been tested directly to the best of our knowledge, functional imaging research has shown activity in the left frontal cortex and inferior frontal gyrus for priming paradigms that require repeated word generation (Buckner et al., 2000).

With regards to the perceptual and conceptual priming distinction in AD, our results showed that there are significant differences between individuals with aMCI/AD dementia and controls on both perceptual and conceptual priming tasks, with no significant difference between these two types of tasks. Unfortunately, the small number of studies that have been conducted in the MCI phase of AD do not allow for follow up analyses. However, we suspect that a distinction between perceptual and conceptual priming can be seen in the MCI phase of AD, in line with earlier findings in aMCI (Gong et al., 2010) but that this distinction disappears as the disease progresses. Future studies should test this hypothesis.

Lastly, the difference in priming between controls and the combined aMCI or AD dementia group was significant in tasks that required deep processing, while it was not significant for tasks that required shallow processing. While the difference in priming between controls and the combined aMCI or AD dementia group was somewhat larger for paradigms that required deep processing than for those that required shallow processing in the exposure phase, the difference between these two task characteristics did not reach significance. One hypothesis for the absence of a significant effect between task characteristics is a lack of statistical power, which is plausible given that the effect size of this moderator is larger than the effect size of the moderator assessing the difference between identification and production priming.

## Additional Considerations, Limitations, and Future Directions

The main complicating factor for the current (or any) meta-analysis is the publication bias that may be present in the literature on repetition priming in AD dementia and aMCI. Specifically, our results suggested that larger effect sizes tended to have a larger standard error. This finding suggests that the overall Hedges’ *g* may be inflated which would mean that the true difference in priming between individuals with aMCI or AD dementia and cognitively healthy older adults could be smaller. Further, there are several factors that can impact reliability which, in turn, can impact difference scores as described by a recent review (Draheim et al., 2019). This recent review also described that defenders of difference scores argue that experimental researchers specifically should not be too concerned about using difference scores so long as group differences consistently emerge. To provide more insight, we reported the priming difference scores in Table 3. While there did not appear to be differences in the *Z*-scores across the task characteristics, it may nonetheless be possible that differences in reliability among priming tasks may have impacted the results. As we did not control for potential differences in the analyses themselves, this does remain a limitation. Another limitation of the current meta-analysis and the state of the literature is that only three studies have assessed priming in an aMCI group directly compared to a cognitively healthy older adult group, which limits our ability to draw conclusions about the impact of paradigm characteristics on priming performance in aMCI, specifically. In addition, the National Institute on Aging—Alzheimer’s Association Research Framework has recently brought forward a neurobiological definition of AD that requires the presence of amyloid deposition, pathologic tau, and neurodegeneration in living persons, which is thought to yield a much less heterogeneous group than previous criteria for AD (Jack et al., 2018). These criteria may allow researchers to better define their sample and may reduce some of the heterogeneity in findings regarding repetition priming in AD. The role that frontally-mediated retrieval deficits play in production priming should also be assessed in future studies.

## Conclusion

The current meta-analysis provides a quantification of the difference between repetition priming between individuals with aMCI or AD. This is, to our knowledge, the first meta-analysis to assess the difference between cognitively healthy older adults and both AD dementia and its earlier stage aMCI. The current meta-analysis extends the literature by exploring task characteristics using moderator analyses. Our results indicated that both individuals with aMCI and AD dementia performed worse on repetition priming tasks than cognitively healthy older adults. Paradigm-related moderators suggested that the Hedges’ *g* between studies comparing the combined aMCI or AD dementia group to cognitively healthy older adults was the highest for paradigms that required participants to produce rather than identify primes during the test phase. Our results further suggested that priming in AD is impaired for both conceptual and perceptual priming tasks. Lastly, while our results suggested that priming in AD is impaired for priming tasks that require deep processing, we were unable to draw firm conclusions about whether the priming deficit is less pronounced in shallow processing in aMCI or AD dementia. Taken together, this meta-analysis helps explain and quantify some of the differences that are present in the literature on repetition priming in aMCI and AD.

## Supplementary Information

Below is the link to the electronic supplementary material.

Supplementary file1 (DOCX 13 KB)

Supplementary file2 (DOCX 13 KB) Supplemental Fig. 1. Flow Diagram. Adapted from PRISMA 2009. ES: Effect Size, k: number of studies

Supplementary file3 (DOCX 30 KB)
