# Supplementary material for: Repetition Priming in Individuals with Amnestic Mild Cognitive Impairment and Alzheimer’s Dementia: a Systematic Review and Meta-Analysis
Source: Neuropsychol Rev. 2021 Apr 25;32(2):228–46. doi: 10.1007/s11065-021-09504-5 (PMC9090892; doi:10.1007/s11065-021-09504-5)
Supplement: Supplementary file 1 — Supplementary file1 (DOCX 13 KB) [file 11065_2021_9504_MOESM1_ESM.docx]

**6. Supplemental Materials**

**Supplement 1. Search Strategy conducted on 04/15/2020.**

**PubMed:** ((("mild cognitive impairment"[tiab] OR "mild cognitive impairments" [tiab] OR "MCI"[tiab] OR "mild neurocognitive disorder"[tiab] OR "mild neurocognitive disorders"[tiab] OR "mild neurocognitive impairment"[tiab]) AND ("Amnesia"[Mesh] OR amnesi*[tiab] OR "Memory"[Mesh:NoExp] OR memory[tiab])) OR ("Alzheimer Disease"[Mesh] OR Alzheimer*[tiab] OR "AD"[tiab])) AND ("Repetition Priming"[Mesh] OR "procedural memory"[tiab] OR "instrumental memory" [tiab] OR "motor memory" [tiab] OR "motor-skill memory" [tiab] OR "muscle memory" [tiab] OR "nondeclarative memory" [tiab] OR "non-declarative memory" [tiab] OR "sequence memory" [tiab] OR "implicit memory" [tiab] OR "habit memory" [tiab] OR "skill memory" [tiab] OR "unconscious memory" [tiab] OR "pattern memory" [tiab] OR "spatial memory" [tiab] OR "spatial memory” [tiab] OR “Spatial memory”[mh] OR “visuospatial memory”[tiab] OR “visuo-spatial memory” [tiab] OR "visuospatial learning" [tiab] OR "visuo-spatial learning" [tiab] OR "procedural learning" [tiab] OR "instrumental learning" [tiab] OR "motor learning" [tiab] OR "motor-skill learning" [tiab] OR ((muscle[tiab] OR muscles[tiab]) AND (learning[tiab] OR memory[tiab])) OR ("Muscles"[Mesh] AND "Learning"[Mesh]) OR "nondeclarative learning" [tiab] OR "non-declarative learning" [tiab] OR "sequence learning" [tiab] OR "implicit learning" [tiab] OR "habit learning" [tiab] OR "skill learning" [tiab] OR "unconscious learning" [tiab] OR "pattern learning" [tiab] OR priming[tiab] OR "skill learning" [tiab] OR "unconscious memory" [tiab] OR "pattern memory" [tiab] OR "spatial memory" [tiab] OR "spatial learning" [tiab] OR “visuospatial memory”[tiab] OR “visuo-spatial memory” [tiab] OR "visuospatial learning" [tiab] OR "visuo-spatial learning" [tiab]) NOT "review"[Publication Type] Filters: Humans; Dutch; English

**PsycINFO:** (((DE "Cognitive Impairment" AND (TI mild OR AB mild)) OR (TI "mild cognitive impairment*" OR "MCI" OR "mild neurocognitive disorder*" OR "mild neurocognitive impairment*" OR "mild neuro-cognitive impairment*") OR (AB "mild cognitive impairment*" OR "MCI" OR "mild neurocognitive disorder*" OR "mild neurocognitive impairment*" OR "mild neuro-cognitive impairment*")) AND (DE "Amnesia" OR DE "Memory Disorders" OR DE "Anterograde Amnesia" OR DE "Global Amnesia" OR DE "Retrograde Amnesia" OR (TI amnes* OR memory) OR (AB amnes* OR memory))) OR (DE "Alzheimer's Disease" OR (TI alzheimer* OR “AD”) OR (AB alzheimer* OR “AD”))) AND ((DE "Priming" OR DE "Implicit Memory" OR DE "Visuospatial Memory" OR DE "Spatial Memory") OR (TI "procedural memory" OR "instrumental memory" OR "motor memory" OR "motor-skill memory" OR "muscle memory" OR "nondeclarative memory" OR "non-declarative memory" OR "sequence memory" OR "implicit memory" OR "habit memory" OR "skill memory" OR "unconscious memory" OR "pattern memory" OR "spatial memory" OR "procedural learning" OR "instrumental learning" OR "motor learning" OR "motor-skill learning” OR “muscle learning” OR "nondeclarative learning" OR "non-declarative learning" OR "sequence learning" OR "implicit learning" OR "habit learning" OR "skill learning” OR "unconscious learning" OR "pattern learning" OR priming OR "Implicit memory" OR “visuospatial learning” OR “visuo-spatial learning” OR “visuospatial memory” OR “visuo-spatial learning”) OR (AB "procedural memory" OR "instrumental memory" OR "motor memory" OR "motor-skill memory" OR "muscle memory" OR "nondeclarative memory" OR "non-declarative memory" OR "sequence memory" OR "implicit memory" OR "habit memory" OR "skill memory" OR "unconscious memory" OR "pattern memory" OR "spatial memory" OR "procedural learning" OR "instrumental learning" OR "motor learning" OR "motor-skill learning” OR “muscle learning” OR "nondeclarative learning" OR "non-declarative learning" OR "sequence learning" OR "implicit learning" OR "habit learning" OR "skill learning" OR "unconscious learning" OR "pattern learning" OR priming OR "Implicit learning" OR “visuospatial learning” OR “visuo-spatial learning” OR “visuospatial memory” OR “visuo-spatial learning”)). Limiters: Publication Type: All Journals, Dissertation Abstract; Language: Dutch, English, Population Group: Human; Methodology: Brain Imaging, Clinical Case Study, Clinical Trial, Empirical Study, Experimental Replication; Follow-up Study; Longitudinal Study, Prospective Study; Retrospective Study; Field Study, Interview. Focus Group Mathematical Model, Non-clinical Case Study, Qualitative Study, Quantitative Study Scientific Simulation. Treatment Outcome, Twin Study
