# Supplementary material for: Repetition Priming in Individuals with Amnestic Mild Cognitive Impairment and Alzheimer’s Dementia: a Systematic Review and Meta-Analysis
Source: Neuropsychol Rev. 2021 Apr 25;32(2):228–46. doi: 10.1007/s11065-021-09504-5 (PMC9090892; doi:10.1007/s11065-021-09504-5)
Supplement: Supplementary file 2 — Supplementary file2 (DOCX 13 KB) Supplemental Fig. 1. Flow Diagram. Adapted from PRISMA 2009. ES: Effect Size, k: number of studies [file 11065_2021_9504_MOESM2_ESM.docx]

**Supplement 3. R-Code**

#turn on required packages

library(metafor)
library(rio)
library(MAd)
library(robumeta)
library(readxl)
#load data
priming <- read_excel("priming.xlsx")
View(priming)
#Assess_publication bias

priming_publication_bias <- rma(measure="SMD", yi=g, vi=vg, weights=weights, data=priming)
summary(priming_publication_bias)

funnel(priming_publication_bias, main = "Funnel Plot")
ranktest(priming_publication_bias)

#run intercept only model

interceptmod <- robu(formula = g ~ 1, data = priming, studynum = studyname, var.eff.size = vg, modelweights = "HIER", small = TRUE)
print (interceptmod)

#create forest plot.

forest.robu(interceptmod, es.lab = "effectsizename",study.lab = "studyname", "Effect Size" = g)

#run models to assess moderators of interest

HierModSm <- robu(formula = g ~ mci_dementia, data = priming, studynum = studynr_new, var.eff.size = vg, modelweights = "HIER", small = TRUE)
print (HierModSm)

HierModSm <- robu(formula = g ~ stimulirepetition data = priming, studynum = studynr_new, var.eff.size = vg, modelweights = "HIER", small = TRUE)
print (HierModSm)

HierModSm <- robu(formula = g ~ perceptual_conceptual, data = priming, studynum = studynr_new, var.eff.size = vg, modelweights = "HIER", small = TRUE)
print (HierModSm)

HierModSm <- robu(formula = g ~ deep_shallow, data = priming, studynum = studynr_new, var.eff.size = vg, modelweights = "HIER", small = TRUE)
print (HierModSm)

HierModSm <- robu(formula = g ~ production_identification, data = priming, studynum = studynr_new, var.eff.size = vg, modelweights = "HIER", small = TRUE)
print (HierModSm)
