# Supplementary material for: Repetition Priming in Individuals with Amnestic Mild Cognitive Impairment and Alzheimer’s Dementia: a Systematic Review and Meta-Analysis
Source: Neuropsychol Rev. 2021 Apr 25;32(2):228–46. doi: 10.1007/s11065-021-09504-5 (PMC9090892; doi:10.1007/s11065-021-09504-5)
Supplement: Supplementary file 3 — Supplementary file3 (DOCX 30 KB) [file 11065_2021_9504_MOESM3_ESM.docx]

Full-text articles assessed for eligibility:
*k* = 276

Records identified through database searching (search conducted on 04/15/2020)
Total *k* = 1591
PubMed *k* = 854
PsycInfo *k* = 737

Records excluded
*k* = 1102

Records screened based on title and abstract
*k* = 1378

Records after removing duplicates
*k* =1378

Articles excluded based on full texts for the following reasons (total k = 244):
No Alzheimer’s or amnestic MCI: *k* = 7
No appropriate reference group: *k* = 6
No Implicit Memory Task: *k* = 20
No Original Work: *k* = 6
No Repetition priming Task or task did not meet outlined criteria: *k* = 159
Insufficient information to calculate ESs: *k* = 44
Dissertations that were later published (i.e. publication was used and dissertation was excluded): *k = 2*

Studies included in quantitative synthesis (meta-analysis)
*k* = 32
